# Reduction of *Arcobacter* at Two Conventional Wastewater Treatment Plants in Southern Arizona, USA

**DOI:** 10.3390/pathogens8040175

**Published:** 2019-10-02

**Authors:** Rajani Ghaju Shrestha, Samendra P. Sherchan, Masaaki Kitajima, Yasuhiro Tanaka, Charles P. Gerba, Eiji Haramoto

**Affiliations:** 1 Interdisciplinary Center for River Basin Environment, University of Yamanashi, 4-3-11 Takeda, Kofu, Yamanashi 400-8511, Japan; rajani_ghaju12@hotmail.com; 2 Division of Sustainable Energy and Environmental Engineering, Osaka University, Suita, Osaka 565-0871, Japan; 3 Department of Global Environmental Health Sciences, Tulane University, 1440 Canal Street, Suite 2100, New Orleans, LA 70112, USA; sshercha@tulane.edu; 4 Division of Environmental Engineering, Hokkaido University, North 13 West 8, Kita-ku, Sapporo, Hokkaido 060-8628, Japan; mkitajima@eng.hokudai.ac.jp; 5 Department of Environmental Sciences, University of Yamanashi, 4-4-37 Takeda, Kofu, Yamanashi 400-8510, Japan; yasuhiro@yamanashi.ac.jp; 6 Water and Energy Sustainable Technology Center, The University of Arizona, Tucson, AZ 85721, USA; gerba@ag.arizona.edu

**Keywords:** *Arcobacter*, next-generation sequencing, virulence gene, wastewater treatment

## Abstract

This study aimed to identify the bacterial community in two wastewater treatment plants (WWTPs) and to determine the occurrence and reduction of *Arcobacter*, along with virulence genes (*ciaB* and *pldA*). A total of 48 samples (24 influent and 24 effluent) were collected at two WWTPs in southern Arizona in the United States, monthly from August 2011 to July 2012. Bacterial DNA extract was utilized for 16S rRNA metagenomic sequencing. Quantification of *Arcobacter* 16S rRNA gene was conducted using a recently developed SYBR Green-based quantitative PCR assay. Among 847 genera identified, 113 (13%) were identified as potentially pathogenic bacteria. *Arcobacter* 16S rRNA gene was detected in all influent samples and ten (83%) and nine (75%) effluent samples at each plant, respectively. Log reduction ratios of *Arcobacter* 16S rRNA gene in Plant A and Plant B were 1.7 ± 0.9 (*n* = 10) and 2.3 ± 1.5 (*n* = 9), respectively. The *ciaB* gene was detected by quantitative PCR in eleven (92%) and twelve (100%) of 12 influent samples from Plant A and Plant B, respectively, while the *pldA* gene was detected in eight (67%) and six (50%) influent samples from Plant A and Plant B, respectively. The prevalence of potentially pathogenic bacteria in WWTP effluent indicated the need for disinfection before discharge into the environment.

## 1. Introduction

Wastewater treatment plants (WWTPs) collect and treat wastewater. Water reclaimed after treatment can be utilized for various purposes, including agricultural irrigation [1,2], recreational purposes [3], to reduce pollution in rivers [4,5], and as a drinking water source [6], as a part of integrated and sustainable water resource management.

*Arcobacter* spp. were first detected in 1991 and are gram-negative, non-spore forming curved or helical rod-shaped bacteria of the family *Campylobacteraceae* [7,8]. *Arcobacter* has been detected at WWTPs in multiple countries, including Canada [9,10], China [11,12,13,14,15], Germany [16], Italy [17], Saudi Arabia [18], South Africa [19], Spain [6,20,21,22,23,24], Turkey [25], the United Kingdom [26], and the United States [27,28,29]. Various types of methods, such as quantitative PCR (qPCR), most probable number-qPCR, multiplex PCR, 16S rDNA restriction fragment length polymorphism, culture, high-throughput sequencing, and fluorescent in situ hybridization [9,10,16,17,18,19], have been adopted in these studies and have demonstrated high abundances of *Arcobacter* spp. in WWTPs. However, most studies have not provided quantitative data on the removal efficiency of *Arcobacter* by WWTPs. There are only a few studies that attempted quantitative detection, but these were limited to only some species of *Arcobacter* or used culture methods [9,18].

Among the studies done at WWTPs in the United States, McLellan et al. (2010) reported a high proportion of *Arcobacter*, along with other bacteria identified by pyrosequencing [28]. Millar and Raghavan (2017) determined the bacterial diversity in WWTP samples and found that *Arcobacter cryaerophilus* contained multiple antibiotic resistant genes and was a major constituent of the sewage microbiome [27]. Sigala and Unc (2013) estimated diversity of antibiotic resistant *Arcobacter* and *Escherichia coli* through pyrosequencing [29]. In Ohio (USA), a waterborne outbreak affected about 1450 persons, and it was believed that groundwater contamination was linked to human waste originating from both wastewater and septic tanks [30]. *Arcobacter*, along with indicator bacteria, pathogenic bacteria, coliphages, and viruses were recovered from associated groundwater wells [30]. Despite various attempts to examine the prevalence, abundance, and persistence of *Arcobacter* as a dominant genus in WWTPs, quantification of this taxon in treatment plants is important for better understanding the incidence of *Arcobacter* and its removal.

In our previous study, a SYBR Green-based qPCR assay targeting a wide range of *Arcobacter* spp. was developed [31]. The designed primers were highly specific to most of the known *Arcobacter* species, could quantify between 1.0 × 10^1^ and 6.4 × 10^6^ copies per reaction, and detect as few as three copies per reaction. This qPCR assay was successful in quantifying the *Arcobacter* 16S rRNA gene in groundwater and surface water samples in Nepal [31].

This study aimed to characterize the bacterial community in the influent and effluent of two WWTPs located in southern Arizona every month over the course of a one-year period. We also investigated the occurrence of *Arcobacter* and associated virulence genes (the invasion gene *ciaB* and the phospholipase gene *pldA*) to determine reduction by two different wastewater treatment processes.

## 2. Results

### 2.1. Characterization of Bacterial Community Using Next-Generation Sequencing (NGS) in Wastewater Samples

**Figure 1:**
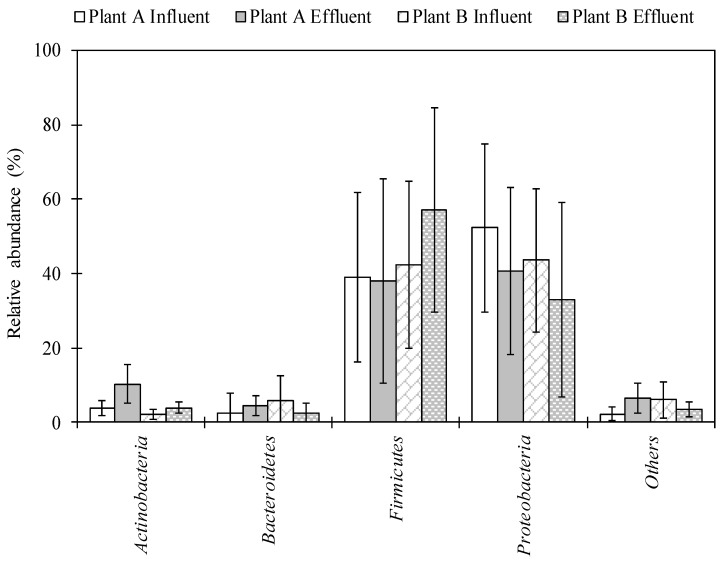
Bacterial composition in wastewater samples of Plant A and Plant B at the phylum level. Bars and error bar represent an average of twelve samples and standard deviation, respectively.

The total numbers of sequences detected using NGS analysis ranged from 4533 to 233,125 and from 60,010 to 272,204 in influent and effluent samples collected in Plant A, respectively. A total of 64,078–262,343 and 63,136–279,359 sequence reads were obtained in influent and effluent samples collected in Plant B, respectively. A total of 29 phyla were detected. *Proteobacteria* and *Firmicutes* were identified in high abundance, ranging from 3.2–89.4% and 4.0–90.8% in Plant A and 3.9–88.9% and 9.6–91.1% in Plant B, respectively (Figure 1). A total of 155–558 and 336–561 genera were identified in the influent and effluent samples of Plant A, respectively. For Plant B, 335–576 and 334–603 genera were identified in the influent and effluent samples, respectively. Among the 847 genera identified, 55 genera, including *Acinetobacter*, *Arcobacter*, *Bacillus*, and *Pseudomonas*, were detected in all influent and effluent wastewater samples from both Plant A and Plant B.

Among the 113 potentially pathogenic bacteria identified, 42 genera were detected at abundances of >1% in at least one of the tested samples (Table 1). In the influent of Plant A, *Bacillus*, *Pseudomonas*, and *Erwinia* were in high abundance (18.6 ± 15.4%, 9.8 ± 24.3%, and 8.5 ± 12.0%, respectively), whereas in effluent samples *Bacillus*, *Mycobacterium*, and *Acinetobacter* were detected in high abundance (25.8 ± 24.7%, 6.5 ± 4.0%, and 3.2 ± 8.0%, respectively). In Plant B, *Bacillus*, *Pseudomonas*, and *Streptococcus* were detected at high abundances (18.9 ± 18.7%, 8.7 ± 22.8%, and 5.1 ± 5.0%, respectively) in influent samples, whereas in effluent samples *Bacillus*, *Pseudomonas*, and *Acinetobacter* were observed to be in high abundances (33.2 ± 25.3%, 5.1 ± 17.3%, and 4.0 ± 11.8%, respectively). There was no significant difference in abundances of most of the potentially pathogenic bacteria between influent and effluent water samples. In Plant A and Plant B, 29 and 34 out of 42 potentially pathogenic bacteria did not show any significant differences in abundance between influent and effluent water samples, respectively (*t*-test, *p* > 0.05).

### 2.2. Occurrence of Total Bacteria, Arcobacter and Associated Virulence Genes in Wastewater Samples

**Figure 2:**
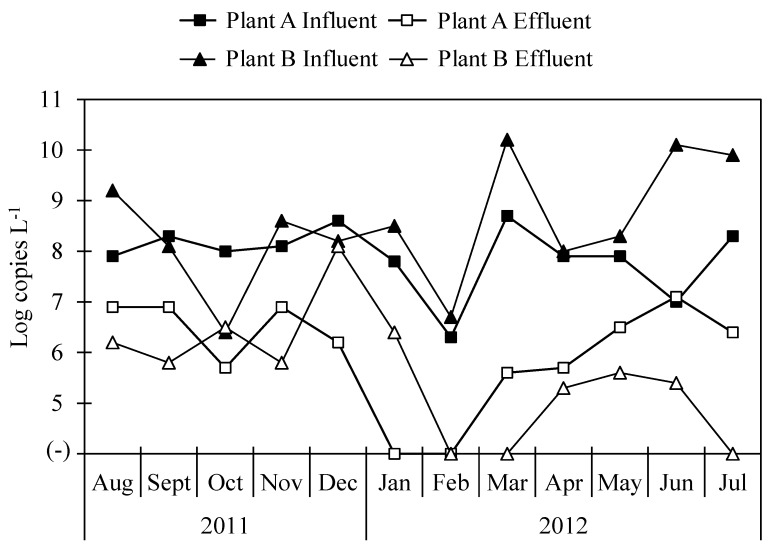
Concentration of *Arcobacter* 16S rRNA gene in influent and effluent samples.

The concentrations of total bacterial 16S rRNA gene in influent samples from both Plant A and Plant B were 9.7 ± 0.3 and 9.7 ± 0.4 log copies L^−1^, respectively, and in effluent samples, the concentrations of total bacterial 16S rRNA gene were 8.0 ± 0.4 and 8.3 ± 0.2 log copies L^−1^ in Plant A and Plant B, respectively. The concentrations of *Arcobacter* 16S rRNA gene in wastewater samples collected from Plant A and Plant B are shown in Figure 2. *Arcobacter* was detected in all influent samples from both Plant A and Plant B and in ten (83%) and nine (75%) of twelve effluent samples at each plant. Average concentrations of *Arcobacter* 16S rRNA gene were 7.9 ± 0.7 and 8.5 ± 1.2 log copies L^−1^ in influent samples of Plant A and Plant B, respectively. Those in effluent samples were 6.4 ± 0.6 and 6.1 ± 0.9 log copies L^−1^ in Plant A and Plant B, respectively.

In our previous study, concentrations of total bacterial 16S rRNA gene obtained using qPCR and abundances of *Arcobacter* obtained using NGS analysis were utilized to compare results obtained from the two methodologies [31]. Here, concentrations of *Arcobacter* 16S rRNA gene were calculated similarly for both Plant A and Plant B. The correlation coefficient of *Arcobacter* 16S rRNA gene concentrations calculated by the two different methods were −0.95 and −0.99 for Plant A and Plant B, respectively.

The *ciaB* gene was detected in eleven (92%) and twelve (100%) of twelve influent samples collected from both Plant A and Plant B, respectively, while the *pldA* gene was detected in eight (67%) and six (50%) influent samples from Plant A and Plant B, respectively. Average concentrations of *ciaB* and *pldA* genes in influent samples were 7.3 ± 0.7 and 5.9 ± 0.2 log copies L^−1^ in Plant A, respectively, whereas those in influent samples were 7.8 ± 1.2 and 6.8 ± 0.8 log copies L^−1^ in Plant B, respectively (Table 2).

### 2.3. Reduction Ratios of Arcobacter and Virulence Genes During Wastewater Treatment

The log reduction ratios of 16S rRNA genes of total bacteria and *Arcobacter* in Plant A, where a conventional activated sludge process is utilized, were 1.6 ± 0.4 (*n* = 12) and 1.7 ± 0.9 (*n* = 10), respectively. Those of total bacteria and *Arcobacter* at Plant B, which utilizes a biological trickling filter process, were 1.5 ± 0.4 (*n* = 12) and 2.3 ± 1.5 (*n* = 9), respectively. For *Arcobacter*, the highest reduction ratios in Plant A and Plant B were obtained in March (3.1 log) and June (4.7 log), respectively. Even though the methodologies for the treatment of wastewater were different between locations, there was no significant difference in log reductions of total bacteria or *Arcobacter* between Plant A and Plant B. The log reduction ratio of the *ciaB* gene was 1.7 ± 1.0 (*n* = 8) and 2.1 ± 1.8 (*n* = 7) in Plant A and Plant B, respectively (Table 3). The log reduction ratio of the *pldA* gene was 1.1 ± 0.1 (*n* = 2) in Plant A.

## 3. Discussion

The overall diversity and abundance of bacterial genera were identified in influent and effluent of wastewater samples using NGS. The phylum *Proteobacteria* was the most abundant phylum, followed by *Firmicutes*, *Bacteroidetes*, and *Actinobacteria* in both WWTPs (Figure 1). These data are in agreement with results of previous studies that tested untreated sewage [27,28] and WWTP samples [29] in the United States. This is also in agreement with the study by Zhang et al. (2012), in which *Proteobacteria* were the most abundant phylum at 14 WWTPs in samples collected from Asia (China, Hong Kong, and Singapore) and North America (Canada and the United States) [32]. Average abundance of *Bacillus* in both plant locations was found to be highest in the effluent (among potentially pathogenic bacteria) (Table 1). Lee et al. (2008) found that concentrations of *Bacillus cereus* were highest in final effluents (disinfected by chlorination and UV radiation) in two WWTPs in Canada [33]. The genus *Pseudomonas* was also abundant and has commonly been detected in wastewater samples collected in the United States [29]. *Pseudomonas* is ubiquitous in the environment, and pathogenic species of *Pseudomonas* can cause infections in hospital patients and/or those with weakened immune systems, such as pneumonia and blood infections [34]. The abundance of *Mycobacterium* significantly increased from influent to effluent samples in Plant A. This taxon has also been found in effluent and activated sludge of a WWTP in Hong Kong via metagenomic analysis [35,36].

The occurrence of potentially pathogenic bacteria in influent and effluent samples emphasizes their ability to persist and be discharged in the environment. WWTPs have been considered a potential hub for evolution and dissemination of antibiotic resistance and virulence genes [37]. Focusing on construction and maintenance of treatment plants, treatment methodologies, and disinfection processes before releasing treated wastewater into the environment will help reduce the spread of potentially pathogenic bacteria into the water environment.

Prevalence of *Arcobacter* has been found to be higher in wastewater compared to other aquatic environments, such as lakes, river, recreational beaches, groundwater, seawater, and drinking water [38]. This is also supported by metagenomic analysis of wastewater samples from various locations where *Arcobacter* has been detected as one of the most abundant genera [19,27,28]. *Arcobacter* is ubiquitous in the environment, and this taxon has been associated with both humans and animals and can cause gastroenteritis, septicemia, mastitis, reproductive disorders, and abortion in livestock [22,39]. *Arcobacter* was detected in all influent samples tested in the current study, and detection in the effluent of both plant locations indicates a high tolerance capability of *Arcobacter*, which can ultimately lead to persistence and spread of the pathogenic bacteria.

Wastewater samples tested in this study have been previously tested for viruses and protozoa. Most of the viruses and protozoa tested did not show any significant differences in log reductions between Plant A and Plant B [40,41]. Similarly, there was no significant difference in the reduction of *Arcobacter* in either Plant A or Plant B. The efficiencies of either plant, operating the activated sludge process or the biological trickling filter process, were not effective in removing pathogens; therefore, they must be improved. When less effectively treated water is used for agricultural or recreational uses, there is a high risk of contamination with *Arcobacter*, as one of the routes of transmission of this taxon is water. Seasonal variations, especially during extreme rainfall, can affect the transportation of *Arcobacter* from WWTPs to groundwater [38]. The concentration of *Arcobacter* was lowest in February but increased in March in Plant A and Plant B. In this study, sample collection for a year may not capture the seasonal variation in *Arcobacter* in WWTPs, and a longer survey period could help gain a better understanding of *Arcobacter* dynamics.

In this study, the *ciaB* gene was detected more frequently than the *pldA* gene in wastewater samples of both plant locations (Table 2). This is likely due to a higher detection frequency for the *ciaB* gene in all *Arcobacter* strains compared to the *pldA* gene [42]. These virulence genes have been detected in *Arcobacter* strains obtained from a variety of species, including humans, chickens, pigs, cattle, sheep, horses, dogs, clams, mussels, and in milk [42,43,44]. These genes have also been identified from isolates of *Arcobacter* recovered from fecal samples originating from humans and from animals [45]. It is evident from these reports that these virulence genes are ubiquitous in the environment. The detection of *ciaB* and *pldA* genes in effluent samples may influence nearby environmental microbial communities, and the dispersal and fate of these genes in water environments suggests negative impacts associated with contaminated wastewater effluent on the gene content of water bodies [46]. The presence of potentially pathogenic organisms and their virulence genes in water demonstrates the importance of monitoring effluent water samples before release into the water bodies and the need for disinfection of the effluent before discharge.

In summary, the characterization of the bacterial community in two WWTPs via NGS detected 113 (13%) of 847 genera as potential pathogenic bacteria. In all influent samples of both plant locations, the *Arcobacter* 16S rRNA gene was quantified and detected in ten and nine of twelve effluent samples at Plant A and Plant B, respectively. There was no significant difference in reduction ratios of the *Arcobacter* 16S rRNA gene between the two plants. Virulence genes, *ciaB* and *pldA*, were also detected in both influent and effluent samples from Plant A and Plant B. The presence of potential pathogenic bacteria and quantification of *Arcobacter* and its virulence genes in effluent samples of WWTPs demonstrate the need for disinfection before discharge into the environment.

## 4. Materials and Methods

### 4.1. Collection of WWTP Samples

As described previously [40], during a 12-month period between August 2011 and July 2012, monthly sampling of influent and effluent wastewater was conducted at two WWTPs in southern Arizona. Wastewater samples from Plants A (conventional activated sludge process) and B (biological trickling filter process) were collected as grab samples. Each sampling was conducted at ~10:00.

### 4.2. Bacterial DNA Extraction

Water samples (100 mL of influent and 1000 mL of effluent) were used to measure viral concentrations using a mixed cellulose ester membrane (pore size: 0.45 µm, diameter: 90 mm; Merck Millipore, Cat. No. HAWP-090-00, Billerica, MA, USA) as described previously [40]. The membrane filter was used for extraction of bacterial DNA after virus elution. In brief, the membrane filter was cut in half, and one piece was mixed with 10 mL of surfactant-based elution buffer in a 50-mL tube. The tube was vortexed vigorously for ~5 min, and the eluate was transferred to a new tube. This step was repeated by adding 5 mL of the elution buffer to the original tube, resulting in ~15 mL of eluate. The tube was centrifuged at 2000× *g* for 10 min at 4 °C, and the supernatant was removed. A volume of 200 µL phosphate buffered saline was added to the tube containing the pellet, mixed, and transferred into a new 2-mL microtube. This step was repeated until the final volume of the bacterial concentrate reached 1 mL. Bacterial DNA (200 μL) was extracted from 200 μL of the concentrate using a QIAamp DNA mini kit (QIAGEN, Hilden, Germany).

### 4.3. NGS for Characterization of Bacterial Communities

The bacterial DNA extracts of wastewater samples were used for metagenomic sequencing via a MiSeq gene sequencer (Illumina, San Diego, CA, USA) as described previously [47]. Operational taxonomic units obtained were analyzed based on the bacterial domain, phylum, family, and genus. A genus was considered as a potentially pathogenic bacterium if any one species of the genus was categorized as biosafety level 2 or 3 by the American Biological Safety Association (https://my.absa.org/tiki-index.php?page=Riskgroups) as described previously [48]. The raw sequences obtained were registered in the NCBI Sequence Read Archive under the accession number PRJNA525124.

### 4.4. qPCR of Total Bacteria and Arcobacter

For total bacterial 16S rRNA gene, qPCR was performed using 515F and U806R primers [49,50] with the thermal conditions, qPCR mixture components, and qPCR reaction conditions as previously described [48]. *Arcobacter* 16S rRNA gene was quantified via qPCR using 2 µL of template DNA, 12.5 µL of a MightyAmp for Real Time (SYBR Plus) (Takara Bio, Kusatsu, Japan), 0.1 µL each of 1 µM of Arco-F and Arco-R-rev primers [31], and 10.3 µL of ultrapure water. For the virulence genes *ciaB* and *pldA*, 2 µL of template DNA, 12.5 µL of a SYBR Premix Ex Taq II (Tli RNase Plus) (Takara Bio), 0.1 µL each of 1 µM of ciaB-F and ciaB-R primers (for the *ciaB* gene) [42] or pldA-F and pldA-R primers (for the *pldA* gene) [42], and 10.3 µL of ultrapure water were used. qPCR was performed with a Thermal Cycler Dice Real Time System Single TP850 (Takara Bio) under the following thermal conditions: for *Arcobacter* 16S rRNA gene, 98 °C for 2 min, followed by 35 cycles at 98 °C for 10 s, 55 °C for 30 s, and 68 °C for 40 s; for the *ciaB* gene, 95 °C for 30 s, followed by 35 cycles at 94 °C for 15 s, 55 °C for 30 s, and 72 °C for 20 s; and for the *pldA* gene, 95 °C for 30s, followed by 35 cycles at 94 °C for 15 s, 56 °C for 45 s, and 72 °C for 20 s. A melting curve analysis was performed to confirm the generation of specific qPCR products.

### 4.5. Statistical Analysis

Student’s *t-*tests were performed with Microsoft Excel 2018 (Microsoft Corporation, Redmond, WA, USA) to determine the difference in abundance of bacteria in influent and effluent wastewater samples. The test was also used to determine whether log reductions of total bacteria and *Arcobacter* at Plants A and B were statistically different. Differences were considered statistically significant if the resulting *p* value was <0.05. The log reduction of *Arcobacter* was calculated from the samples, which were qPCR-positive for both influent and effluent samples.

## Figures and Tables

**Table 1:** Abundance ratios of potential pathogenic bacteria in influent and effluent water samples from Plant A and Plant B.

Potential Pathogenic Genus	Plant A			Plant B		
	Influent (Average ± s.d.)	Effluent (Average ± s.d.)	*p-*Value	Influent (Average ± s.d.)	Effluent (Average ± s.d.)	*p*-Value
*Achromobacter*	0.2 ± 0.4	0.1 ± 0.1	0.35	0.1 ± 0.3	0.0 ± 0.1	0.42
*Acidovorax*	0.9 ± 1.5	2.8 ± 2.9	0.06	2.0 ± 1.7	1.1 ± 1.5	0.15
*Acinetobacter*	6.2 ± 11.8	3.2 ± 8.0	0.48	1.7 ± 1.6	4.0 ± 11.8	0.52
*Actinobacillus*	0.1 ± 0.1	0.1 ± 0.2	0.40	1.3 ± 1.8	0.0 ± 0.1	**0.04**
*Arcobacter*	1.0 ± 1.0	0.5 ± 0.5	0.21	4.1 ± 5.7	0.9 ± 2.5	0.10
*Bacillus*	18.6 ± 15.4	25.8 ± 24.7	0.40	18.9 ± 18.7	33.2 ± 25.3	0.13
*Bacteroides*	0.1 ± 0.2	0.1 ± 0.2	0.58	1.6 ± 2.1	0.2 ± 0.4	**0.04**
*Bifidobacterium*	0.8 ± 0.5	0.7 ± 0.4	0.36	1.0 ± 0.6	1.3 ± 0.9	0.26
*Blautia*	2.4 ± 2.1	0.4 ± 0.4	**0.01**	2.5 ± 1.8	1.4 ± 3.1	0.34
*Brevundimonas*	0.2 ± 0.5	0.1 ± 0.1	0.22	0.1 ± 0.2	0.0 ± 0.1	0.57
*Chromobacterium*	<0.01	0.5 ± 0.7	0.05	0.1 ± 0.1	0.1 ± 0.1	0.46
*Chryseobacterium*	1.0 ± 2.0	1.1 ± 1.1	0.94	1.5 ± 1.6	0.5 ± 0.8	0.08
*Clostridium*	0.3 ± 0.2	0.4 ± 0.2	0.68	0.5 ± 0.3	0.4 ± 0.3	0.19
*Comamonas*	0.5 ± 0.4	0.2 ± 0.2	**0.03**	1.1 ± 0.8	0.4 ± 0.4	**0.03**
*Eikenella*	0.6 ± 0.5	0.1 ± 0.1	**0.00**	0.6 ± 0.5	0.2 ± 0.4	0.06
*Enterobacter*	1.6 ± 1.3	0.4 ± 0.9	**0.03**	0.6 ± 0.6	1.7 ± 3.8	0.37
*Enterococcus*	0.5 ± 0.4	0.1 ± 0.1	**0.00**	0.3 ± 0.4	0.2 ± 0.2	0.13
*Erwinia*	8.5 ± 12.0	<0.01	**0.03**	3.3 ± 5.6	0.5 ± 0.9	0.10
*Flavobacterium*	0.1 ± 0.1	0.8 ± 0.8	**0.00**	0.4 ± 0.8	0.2 ± 0.3	0.41
*Klebsiella*	0.1 ± 0.1	0.1 ± 0.1	0.69	0.3 ± 0.4	0.1 ± 0.3	0.38
*Gordonia*	0.1 ± 0.0	0.6 ± 1.6	0.32	0.0 ± 0.1	0.2 ± 0.2	**0.01**
*Lactococcus*	0.3 ± 0.6	<0.01	0.13	<0.01	<0.01	0.14
*Leptotrichia*	0.2 ± 0.3	0.5 ± 0.5	0.11	0.8 ± 1.0	0.1 ± 0.2	**0.03**
*Megasphaera*	0.1 ± 0.1	0.1 ± 0.1	0.85	0.4 ± 0.3	0.2 ± 0.3	0.17
*Microbacterium*	0.8 ± 0.8	0.3 ± 0.4	**0.04**	0.1 ± 0.1	0.1 ± 0.1	0.86
*Mycobacterium*	0.2 ± 0.2	6.5 ± 4.0	**0.00**	0.1 ± 0.0	0.1 ± 0.1	0.06
*Neisseria*	0.9 ± 1.0	0.5 ± 0.7	0.15	2.3 ± 2.8	0.2 ± 0.5	**0.03**
*Paenibacillus*	0.4 ± 0.4	2.7 ± 3.1	**0.02**	0.5 ± 1.3	2.7 ± 3.2	**0.04**
*Parabacteroides*	0.0 ± 0.1	<0.01	0.95	0.5 ± 0.6	0.0 ± 0.1	**0.04**
*Paracoccus*	0.6 ± 0.6	0.2 ± 0.4	0.09	0.3 ± 0.3	0.4 ± 0.7	0.65
*Plesiomonas*	0.1 ± 0.1	0.1 ± 0.1	0.54	0.7 ± 1.1	0.2 ± 0.4	0.15
*Prevotella*	<0.01	<0.01	0.21	0.5 ± 0.7	0.0 ± 0.1	0.06
*Pseudomonas*	9.8 ± 24.3	0.1 ± 0.2	0.19	8.7 ± 22.8	5.1 ± 17.3	0.67
*Psychrobacter*	0.4 ± 0.5	0.0 ± 0.1	0.08	<0.01	0.1 ± 0.1	0.62
*Rhodococcus*	0.7 ± 0.5	<0.01	**0.00**	0.2 ± 0.2	0.1 ± 0.1	0.18
*Sebaldella*	0.1 ± 0.1	0.0 ± 0.0	0.05	0.7 ± 1.2	0.1 ± 0.3	0.13
*Sphingobacterium*	0.6 ± 1.9	0.1 ± 0.1	0.34	<0.01	0.1 ± 0.1	0.31
*Stenotrophomonas*	1.7 ± 4.7	0.8 ± 1.9	0.56	0.3 ± 0.4	0.6 ± 1.3	0.41
*Streptococcus*	6.4 ± 4.1	1.1 ± 0.8	**0.00**	5.1 ± 5.0	2.4 ± 4.2	0.17
*Sutterella*	<0.01	1.7 ± 1.8	**0.01**	0.0 ± 0.1	<0.01	0.16
*Veillonella*	0.1 ± 0.1	0.1 ± 0.1	0.86	0.4 ± 0.5	0.2 ± 0.2	0.20
*Yersinia*	0.2 ± 0.5	0.2 ± 0.4	0.89	0.8 ± 2.1	1.0 ± 2.5	0.84

s.d.: standard deviation. Letters in bold represent *p* values < 0.05.

**Table 2:** Concentrations of *ciaB* and *pldA* genes of *Arcobacter* in influent and effluent samples.

Time of Sample Collection		Plant A (Log Copies L^−1^)				Plant B (Log Copies L^−1^)			
		*ciaB*		*pldA*		*ciaB*		*pldA*	
		Influent	Effluent	Influent	Effluent	Influent	Effluent	Influent	Effluent
2011	August	7.3	6.2	5.7	4.5	8.5	5.1	6.5	n.d.
	September	7.8	5.8	5.8	n.d.	7.1	4.4	5.7	n.d.
	October	7.9	5.1	5.9	n.d.	5.4	6.0	n.d.	n.d.
	November	7.7	6.2	5.7	n.d.	8.1	n.d.	n.d.	n.d.
	December	8.0	5.6	5.8	4.8	7.9	7.7	n.d.	5.3
2012	January	7.4	n.d.	n.d.	n.d.	8.1	6.3	n.d.	5.1
	February	6.4	n.d.	n.d.	n.d.	5.7	n.d.	n.d.	n.d.
	March	7.4	n.d.	5.9	n.d.	8.6	n.d.	7.2	n.d.
	April	n.d.	n.d.	n.d.	n.d.	6.9	4.6	n.d.	n.d.
	May	6.9	5.2	5.9	n.d.	7.0	n.d.	6.0	n.d.
	June	5.7	6.2	n.d.	5.0	9.3	4.6	7.7	n.d.
	July	7.7	5.4	6.2	n.d.	9.1	n.d.	7.5	n.d.
Mean ± s.d.		7.3 ± 0.7	5.7 ± 0.5	5.9 ± 0.2	4.8 ± 0.3	7.6 ± 1.2	5.5 ± 1.2	6.8 ± 0.8	5.2 ± 0.1

s.d.: standard deviation; n.d.: not detected.

**Table 3:** Reduction of total bacteria and *Arcobacter* at WWTPs.

Log Reduction Tested	Log Reduction (mean ± s.d.)	
	Plant A	Plant B
16S rRNA gene of total bacteria	1.6 ± 0.4 (*n* = 12)	1.5 ± 0.4 (*n* = 12)
16S rRNA gene of *Arcobacter*	1.7 ± 0.9 (*n* = 10)	2.3 ± 1.5 (*n* = 9)
*ciaB* gene of *Arcobacter*	1.7 ± 1.0 (*n* = 8)	2.1 ± 1.8 (*n* = 7)
*pldA* gene of *Arcobacter*	1.1 ± 0.1 (*n* = 2)	n.d.

s.d.: standard deviation; n.d.: not determined.
